# Macrolide susceptibility and serotype specific macrolide resistance of invasive isolates of *Streptococcus pneumoniae *in Germany from 1992 to 2008

**DOI:** 10.1186/1471-2180-10-299

**Published:** 2010-11-25

**Authors:** Matthias Imöhl, Ralf René Reinert, Christina Mutscher, Mark van der Linden

**Affiliations:** 1 National Reference Center for Streptococci, Department of Medical Microbiology, University Hospital RWTH Aachen, Pauwelsstrasse 30, D-52074 Aachen, Germany; 2 Department of Medical Statistics, University Hospital RWTH Aachen, Pauwelsstrasse 30, D-52074 Aachen, Germany; 3 Wyeth Vaccines Research, Paris La Défense, Paris, France

## Abstract

**Background:**

Macrolide resistant *Streptococcus pneumoniae *has been on a gradual increase in Germany for over a decade. The current study was undertaken against the background of the recent observation of declining macrolide resistance rates especially among German children. Nationwide surveillance of invasive pneumococcal disease has been conducted in Germany since 1992. A population- and laboratory-based approach was used to collect data on invasive pneumococcal disease, and isolates sent to the National Reference Center for Streptococci by diagnostic microbiological laboratories from 1992 to 2008 were included in this study.

**Results:**

From 1992 to 2008, data on macrolide susceptibility were available for 11,807 invasive isolates. 8,834 isolates (74.8%) were from adults (≥ 16 years), and 2,973 isolates (25.2%) from children (< 16 years). The overall nonsusceptibility rate of all isolates was 16.2% (intermediate, 0.2%; resistant, 16.0%). Higher resistance rates were observed among children (intermediate, 0.2%; resistant, 23.8%) than among adults (intermediate, 0.3%; resistant 13.4%). Maximum nonsusceptibility rates during the period under study were observed in 2005 (children: intermediate, 0.3%; resistant, 32.3%; adults: intermediate, 0.0%; resistant, 18.6%), while nonsusceptibility rates in 2008 were considerably lower, especially for children (children: intermediate, 0.0%; resistant, 15.2%; adults: intermediate, 0.1%; resistant, 12.9%). The rate of resistance was higher among the vaccine serotypes (7-valent, 36.6%; 10-valent, 28.2%; 13-valent, 24.3%) than among the non vaccine serotypes (non 7-valent, 6.5%; non 10-valent, 7.4%; non 13-valent, 6.3%). Serotype 14 (69.6% nonsusceptibility) proved to be the most resistant serotype.

**Conclusions:**

There has been a considerable and statistically significant decrease in macrolide nonsusceptibility in Germany since 2005, especially among children.

## Background

*Streptococcus pneumoniae *is a leading pathogen in bacterial pneumonia, sepsis and meningitis in humans worldwide [1,2]. In many European countries the rate of resistance of *S. pneumoniae *to macrolides has exceeded that of penicillin [3]. Concerning penicillin, it has been described that treatment of patients with nonmeningeal invasive pneumococcal infections with nonsusceptible isolates was not associated with higher mortality rates [4-6]. In 2008 new penicillin breakpoints for *S. pneumoniae *were published by the CLSI [7], differentiating meningitis and non-meningitis cases of invasive pneumococcal disease (IPD). Their impact on susceptibility categorisation in Germany was described previously by our group [8]. However, for macrolides an increased risk of macrolide failure has been reported for pneumococcal isolates nonsusceptible in vitro [9].

The aim of this study was to evaluate macrolide susceptibility of all isolates of *S. pneumoniae *with IPD that were sent to the German National Reference Center for Streptococci (NRCS) between 1992 and 2008 and to evaluate potential trends in nonsusceptibility over time. The description of serotype specific resistance, was a major aim of the study.

The study was undertaken against the background of the recent observation of declining macrolide resistance rates especially among German children.

## Methods

### Study design

The NRCS has conducted surveillance for invasive pneumococcal disease in Germany since 1992. A population- and laboratory-based approach was used to collect data on invasive pneumococcal disease among children < 16 years and adults ≥ 16 years in Germany. Isolates were sent to the NRCS by diagnostic microbiological laboratories throughout Germany on a voluntary basis. Cases from January 1, 1992 to December 31, 2008 were included in this study. A case of IPD was defined by the isolation of *S. pneumoniae *from a normally sterile site.

### Microbiological investigations

Isolates were identified by standard procedures including bile solubility and optochin sensitivity. Minimal inhibitory concentrations (MIC) testing was performed using the broth microdilution method as recommended by the Clinical and Laboratory Standards Institute (CLSI) [7]. Macrolide resistance was investigated using erythromycin or clarithromycin, in which testing with erythromycin was replaced by clarithromycin over the years. 425 isolates were tested both for erythromycin and clarithromycin. The susceptible, intermediate, and resistant breakpoints (MIC) were ≤ 0.25, 0.5, and ≥1 μg/ml, both for erythromycin and clarithromycin [7]. *Streptococcus pneumoniae *ATCC 49619 was used as a control strain.

### Statistical analysis

All categorical data were expressed as frequencies. To analyse a severe increase or decrease over time the Cochran-Armitage test was used. The overall significance level was adjusted using the Bonferroni correction to account for the problem of multiple testing. Due to 14 tests p-values ≤ 0.0036 were considered as statistically significant test results. All statistical analyses were conducted using SAS Version 9.1.3 (SAS Institute Inc., Cary, NC, USA).

## Results

In total, 12,136 isolates from invasive pneumococcal disease were collected between January 1, 1992 and December 31, 2008. The number of cases for each year vary between 297 and 2,037 (median: 505 cases). Data on macrolide susceptibility were available for 11,807 isolates, whereas 8,834 isolates (74.8%) originated from adults, 2,973 isolates (25.2%) were from children.

The overall nonsusceptibility rate of all isolates was 16.2% (intermediate, 0.2%; resistant, 16.0%). Higher resistance rates were observed among children (intermediate, 0.2%; resistant, 23.8%) than among adults (intermediate, 0.3%; resistant 13.4%) (Table 1).

**Table 1:** Ranking of serotype specific macrolide nonsusceptibility among IPD isolates in Germany from 1992 to 2008 (n, overall = 11,807; n, adults = 8,834; n, children = 2,973)

	children				adults				overall				
**Sero** **type**	**I%**	**R%**	**S%**	**total** **(n)**	**I%**	**R%**	**S%**	**total** **(n)**	**I%**	**R%**	**S%**	**total**^†^ **(n)**	**total**^‡^ **(%)**

14	0.0	67.4	32.6	663	0.2	71.0	28.8	883	0.1	69.5	30.4	1546	16.4
45	-	-	-	-	0.0	33.3	66.7	3	0.0	33.3	66.7	3	0.0
19B	0.0	0.0	100.0	1	0.0	50.0	50.0	2	0.0	33.3	66.7	3	0.0
rough	0.0	25.0	75.0	8	0.0	40.0	60.0	10	0.0	33.3	66.7	18	0.2
6B	0.0	29.3	70.7	215	0.4	36.2	63.4	232	0.2	32.9	66.9	447	4.8
15A	4.8	28.6	66.7	21	0.0	33.3	66.7	27	2.1	31.3	66.7	48	0.5
19F	0.0	24.5	75.5	212	0.4	27.5	72.0	236	0.2	26.1	73.7	448	4.8
19A	0.0	24.4	75.6	90	0.9	26.0	73.2	231	0.6	25.5	73.8	321	3.4
10B	-	-	-	-	0.0	20.0	80.0	10	0.0	20.0	80.0	10	0.1
19C	0.0	0.0	100.0	2	0.0	33.3	66.7	3	0.0	20.0	80.0	5	0.1
15B	0.0	23.1	76.9	26	0.0	17.5	82.5	57	0.0	19.3	80.7	83	0.9
23F	0.5	20.4	79.1	201	0.6	18.3	81.2	356	0.5	19.0	80.4	557	5.9
9V	0.9	13.2	85.8	106	1.3	20.1	78.5	298	1.2	18.3	80.4	404	4.3
NT	0.0	10.0	90.0	10	0.0	20.7	79.3	29	0.0	17.9	82.1	39	0.4
11F	-	-	-	-	0.0	16.7	83.3	6	0.0	16.7	83.3	6	0.1
15C	0.0	15.4	84.6	26	0.0	14.8	85.2	27	0.0	15.1	84.9	53	0.6
9A	0.0	9.5	90.5	21	0.0	19.2	80.8	26	0.0	14.9	85.1	47	0.5
33B	0.0	0.0	100.0	3	0.0	25.0	75.0	4	0.0	14.3	85.7	7	0.1
33A	0.0	11.1	88.9	9	0.0	14.3	85.7	21	0.0	13.3	86.7	30	0.3
33F	0.0	0.0	100.0	17	0.0	17.6	82.4	51	0.0	13.2	86.8	68	0.7
12B	0.0	0.0	100.0	3	0.0	20.0	80.0	5	0.0	12.5	87.5	8	0.1
6A	0.0	5.5	94.5	128	0.4	9.7	89.9	277	0.2	8.4	91.4	405	4.3
28A	0.0	0.0	100.0	4	0.0	12.5	87.5	8	0.0	8.3	91.7	12	0.1
35F	0.0	10.0	90.0	10	0.0	7.8	92.2	64	0.0	8.1	91.9	74	0.8
24F	0.0	6.8	93.2	44	0.0	6.9	93.1	72	0.0	6.9	93.1	116	1.2
13	0.0	0.0	100.0	3	0.0	8.3	91.7	12	0.0	6.7	93.3	15	0.2
16F	0.0	0.0	100.0	7	3.7	7.4	88.9	27	2.9	5.9	91.2	34	0.4
17F	0.0	12.5	87.5	8	0.0	3.2	96.8	31	0.0	5.1	94.9	39	0.4
38	0.0	0.0	100.0	23	0.0	7.9	92.1	38	0.0	4.9	95.1	61	0.6
34	0.0	16.7	83.3	6	0.0	0.0	100.0	15	0.0	4.8	95.2	21	0.2
9N	0.0	0.0	100.0	25	0.0	5.5	94.5	145	0.0	4.7	95.3	170	1.8
11A	0.0	0.0	100.0	15	0.0	5.2	94.8	135	0.0	4.7	95.3	150	1.6
18A	0.0	0.0	100.0	10	0.0	8.3	91.7	12	0.0	4.5	95.5	22	0.2
1	0.4	5.2	94.4	232	0.2	3.5	96.3	458	0.3	4.1	95.7	690	7.3
7F	0.0	3.9	96.1	203	0.4	3.7	95.9	515	0.3	3.8	96.0	718	7.6
5	0.0	0.0	100.0	19	0.0	5.4	94.6	37	0.0	3.6	96.4	56	0.6
10A	0.0	4.0	96.0	50	0.0	2.5	97.5	122	0.0	2.9	97.1	172	1.8
4	0.0	2.9	97.1	102	0.0	2.2	97.8	409	0.0	2.3	97.7	511	5.4
20	0.0	0.0	100.0	5	0.0	2.6	97.4	38	0.0	2.3	97.7	43	0.5
18C	0.6	1.7	97.8	181	0.0	2.8	97.2	145	0.3	2.1	97.5	326	3.5
3	0.0	3.1	96.9	96	0.2	1.8	98.0	663	0.1	2.0	97.9	759	8.1
12F	0.0	0.0	100.0	16	0.0	1.9	98.1	105	0.0	1.7	98.3	121	1.3
8	0.0	0.0	100.0	18	0.5	1.6	97.9	190	0.5	1.4	98.1	208	2.2
23A	0.0	0.0	100.0	14	0.0	1.4	98.6	74	0.0	1.1	98.9	88	0.9
22F	0.0	0.0	100.0	20	0.5	0.5	98.9	186	0.5	0.5	99.0	206	2.2
2	0.0	0.0	100.0	1	0.0	0.0	100.0	11	0.0	0.0	100.0	12	0.1
31	0.0	0.0	100.0	1	0.0	0.0	100.0	25	0.0	0.0	100.0	26	0.3
12A	0.0	0.0	100.0	3	0.0	0.0	100.0	9	0.0	0.0	100.0	12	0.1
18F	0.0	0.0	100.0	5	0.0	0.0	100.0	10	0.0	0.0	100.0	15	0.2
23B	0.0	0.0	100.0	6	0.0	0.0	100.0	11	0.0	0.0	100.0	17	0.2
35B	0.0	0.0	100.0	3	0.0	0.0	100.0	8	0.0	0.0	100.0	11	0.1
9L	0.0	0.0	100.0	5	0.0	0.0	100.0	12	0.0	0.0	100.0	17	0.2
Others*	0.0	0.0	100.0	31	0.0	0.0	100.0	62	0.0	0.0	100.0	93	1.0
not serotyped	0.0	4.4	95.6	45	0.2	0.0	99.8	2360	0.2	0.1	99.8	2405	-
total (%)	0.2	23.8	76.1	-	0.3	13.4	86.3	-	0.2	16.0	83.7	-	100.0
total (n)	5	707	2261	2973	24	1184	7626	8834	29	1891	9887	11807	9402

I%, intermediate isolates in percent; R%, resistant isolates in percent; S%, susceptible isolates in percent; n, number of isolates tested.

Total (n)^† ^represents the number of isolates collected from both children and adults. Total (%)^‡ ^represents the serotype distribution in percent in relation to the number of 9402 IPD isolates serotyped.

Others* includes the serotypes (number of isolates, overall): 15F (9), 18B (9), 7C (8), 10F (6), 11B (6), 35A (6), 29 (5), 21 (4), 24A (4), 28F (4), 35C (4), 37 (4), 22A (3), 36 (3), 7A (3), 24B (2), 25F (2), 39 (2), 48 (2), 7B (2), 17A (1), 18 (1), 19 (1), 35 (1), 9 (1).

The sampling source related nonsusceptibility is shown in Table 2. Highest nonsusceptibility rates were observed for pharyngeal isolates (75%, n = 4), pericardium (50%, n = 8) and mastoid (40%, n = 10). Nonsusceptibility rates for CSF and blood were 17.8% (n = 1824) and 15.9% (n = 9352), respectively. The serotype distribution broken down to the sampling source is shown in Table 3. For blood, CSF and BAL serotype 14 is most prevalent, whereas for pleural fluid serotypes 1 and 3 are most often found.

**Table 2:** Ranking of macrolide nonsusceptibility among IPD isolates in Germany from 1992 to 2008 related to the sampling source (n = 11,807)

Sampling source	I%	R%	I+R%	total (n)
Pharynx	0.0	75.0	**75.0**	4
Pericard	0.0	50.0	**50.0**	8
Mastoid	0.0	40.0	**40.0**	10
BAL	0.6	18.7	**19.4**	154
Others/unknown	0.0	18.3	**18.3**	131
CSF	0.2	17.7	**17.8**	1824
Blood	0.3	15.6	**15.9**	9352
Pleural fluid	0.4	14.7	**15.1**	252
Eye	0.0	11.1	**11.1**	9
Ascites	0.0	8.7	**8.7**	23
Joint	0.0	5.6	**5.6**	36
Ear	0.0	0.0	**0.0**	4

**Total**	**0.3**	**16.0**	**16.3**	**11807**

**Table 3:** Serotype distribution among IPD isolates from different sampling sites in Germany from 1992 to 2008 in percent (n = 11,807)

Sero type	Ascites	BAL	Blood	CSF	Joint	Pleural fluid	Total (%)	Total (n)
14	9,1	10,7	17,4	14,8	0,0	11,0	16,5	1546
3	0,0	6,0	8,6	5,7	3,0	13,8	8,1	759
7F	4,5	1,2	8,0	7,2	0,0	7,2	7,7	718
1	4,5	6,0	8,3	2,6	12,1	15,5	7,4	690
23F	4,5	8,3	5,7	7,1	3,0	6,6	5,9	557
4	4,5	7,1	6,0	3,8	6,1	3,9	5,5	511
19F	9,1	7,1	4,2	6,8	3,0	3,9	4,8	448
6B	9,1	6,0	4,3	6,6	12,1	4,4	4,8	447
6A	4,5	2,4	4,0	5,7	12,1	4,4	4,3	405
9V	0,0	4,8	4,8	2,3	6,1	2,8	4,3	404
18C	4,5	3,6	2,8	5,8	9,1	3,3	3,5	326
19A	4,5	4,8	3,5	2,7	3,0	1,7	3,4	321
8	9,1	1,2	2,4	2,0	0,0	0,6	2,2	208
22F	0,0	0,0	2,3	2,0	6,1	0,6	2,2	206
10A	4,5	1,2	1,6	2,7	3,0	1,7	1,8	172
9N	0,0	2,4	1,9	1,7	0,0	1,1	1,8	170
11A	0,0	1,2	1,6	1,7	0,0	2,8	1,6	150
12F	4,5	2,4	1,3	1,2	0,0	0,6	1,3	121
24F	0,0	0,0	1,2	1,6	0,0	0,6	1,2	116
23A	0,0	1,2	0,8	1,2	0,0	2,8	0,9	88
15B	0,0	0,0	0,7	1,5	3,0	1,7	0,9	83
35F	4,5	0,0	0,7	1,0	0,0	1,7	0,8	74
33F	0,0	1,2	0,6	1,2	3,0	0,6	0,7	68
38	0,0	0,0	0,6	0,8	0,0	0,0	0,7	61
5	0,0	0,0	0,7	0,3	0,0	0,6	0,6	56
15C	4,5	1,2	0,5	0,7	3,0	0,0	0,6	53
15A	0,0	0,0	0,5	0,7	0,0	1,1	0,5	48
9A	0,0	1,2	0,5	0,4	0,0	1,1	0,5	47
20	0,0	0,0	0,4	0,5	0,0	1,1	0,5	43
17F	4,5	3,6	0,3	0,6	0,0	0,0	0,4	39
NT	0,0	2,4	0,4	0,3	3,0	0,0	0,4	39
16F	0,0	0,0	0,3	0,6	0,0	0,6	0,4	34
33A	0,0	0,0	0,3	0,4	0,0	0,6	0,3	30
31	0,0	2,4	0,3	0,2	0,0	0,0	0,3	26
18A	0,0	0,0	0,2	0,4	3,0	0,0	0,2	22
34	0,0	0,0	0,1	0,6	0,0	0,6	0,2	21
Others*	9,1	10,7	2,3	4,5	6,1	1,7	2,8	264
Total	100,0	100,0	100,0	100,0	100,0	100,0	100,0	9371

not serotyped	4,3	45,5	23,8	2,1	8,3	28,2	20,6	2436

Only sampling sites with ≥ 20 isolates were included in this table.

NT: nontypeable; n: number of isolates tested.

Others* includes the serotypes (number of isolates): rough (18), 9L (17), 23B (17), 13 (15), 18F (15), 12A (12), 2 (12), 28A (12), 35B (11), 10B (10), 15F (9), 18B (9), 12B (8), 33B (7), 7C (6), 10F (6), 11B (6), 11F (6), 35A (6), 7A (5), 19C (5), 29 (5), 21 (4), 24A (4), 28F (4), 35C (4), 37 (4), 19B (3), 22A (3), 36 (3), 45 (3), 7B (2), 24B (2), 25F (2), 39 (2), 48 (2), 9(1), 17A (1), 18 (1), 19 (1), 35 (1).

**Figure 1:**
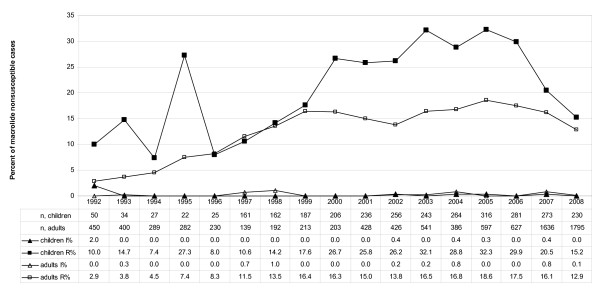
**Macrolide nonsusceptibilities of IPD isolates in Germany**. Macrolide nonsusceptibilities of IPD isolates in Germany (1992 to 2008; n, total = 11,807; n, adults = 8,834; n, children = 2,973; I%, intermediate in percent; R%, resistant in percent; n, number of cases).

**Figure 2:**
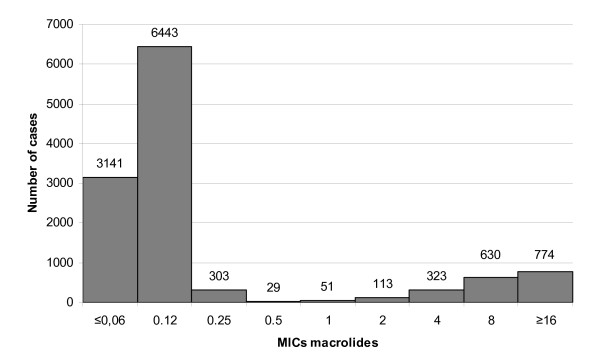
**Minimum inhibitory concentrations (MICs) of invasive isolates**. Minimum inhibitory concentrations (MICs) of invasive isolates (1992-2008, n = 11,807)

As for the childhood IPD isolates in the first year of this study (1992), 2.0% were intermediate and 10.0% resistant to macrolides. Maximum nonsusceptibility rates during the period under study were observed in 2005 (intermediate, 0.3%; resistant, 32.3%), while in 2008, 0.0% of isolates were intermediate and 15.2% resistant. IPD isolates obtained from adults were intermediate in 0.0% and resistant in 2.9% in 1992. Maximum nonsusceptibility rates were observed in 2005 as well (intermediate, 0.0%; resistant, 18.6%). Nonsusceptibility rates in 2008 were 0.1% (intermediate) and 12.9% (resistant). The increase in macrolide nonsusceptibility from 1992 to 2005 was statistically significant for children (P < 0.0001) and adults (P < 0.0001), as well as the decrease from 2005 to 2008 (children, P < 0.0001; adults, P < 0.0001). Concerning the intermediate resistant isolates no significant trends were observed (1992-2005: children (P = 0.8942), adults (P = 0.4302); 2005-2008: children (P = 0.6282), adults (P = 0.5960)). Detailed results of the macrolide susceptibility testing are shown in Figure 1. The MICs of all invasive isolates are illustrated in Figure 2.

Overall, the leading serotypes were serotypes 14 (16.4% of serotyped isolates), 3 (8.1%), 7F (7.6%), 1 (7.3%) and 23F (5.9%). A ranking of serotype specific macrolide nonsusceptibility of IPD isolates is shown in Table 1. Serotype 14 (69.5% nonsusceptibility) was by far the most resistant serotype, followed by serotypes rough, 19B, 45 (33.3% each), 6B (32.9%), 15A (31.3%), 19F (26.1%), and 19A (25.5%). However, absolute numbers for rough, 19B and 45 were very low.

**Figure 3:**
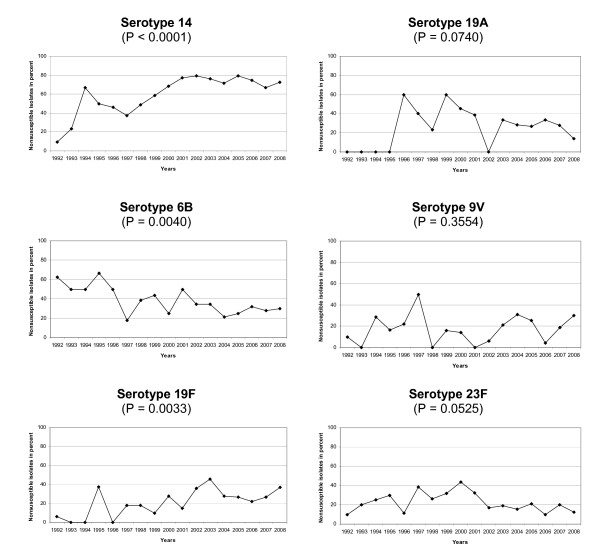
**Serotype specific macrolide nonsusceptibility of IPD isolates in Germany**. Serotype specific macrolide nonsusceptibility of IPD isolates in Germany (1992 to 2008; n, serotype 14 = 1,546; n, serotype 6B = 447; n, serotype 19F = 448; n, serotype 19A = 321; n, serotype 9V = 404; n, serotype 23F = 557)

Serotypes contributing considerably to pneumococcal macrolide nonsusceptibility by combination of frequency among invasive isolates and relatively high macrolide nonsusceptibility are especially serotypes 14, 6B, 19F, 19A, 9V and 23F. The development of nonsusceptibility of these serotypes over the years is shown in Figure 3. The nonsusceptibility among serotype 14 isolates increases considerably over the years up to around 80% (P < 0.0001). For serotype 19F a significant increase (P = 0.0033) in nonsusceptibility was observed as well. No significant trends were found for serotypes 6B (P = 0.0040), 9V (P = 0.3554), 19A (P = 0.0740) and 23F (P = 0.0529).

**Figure 4:**
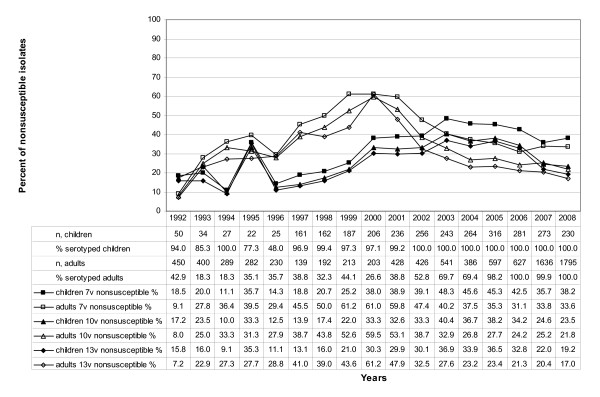
**Macrolide nonsusceptibility among 7-, 10- and 13-valent vaccine serotypes**. Macrolide nonsusceptibility among 7-, 10- and 13-valent vaccine serotypes (IPD isolates in Germany from 1992 to 2008; n, number of cases. Vaccine strains included are: 7-valent: serotypes 4, 6B, 9V, 14, 18C, 19F and 23F; 10-valent: 7-valent serotypes plus 1, 5 and 7F; 13-valent: 10-valent serotypes plus 3, 19A and 6A)

**Figure 5:**
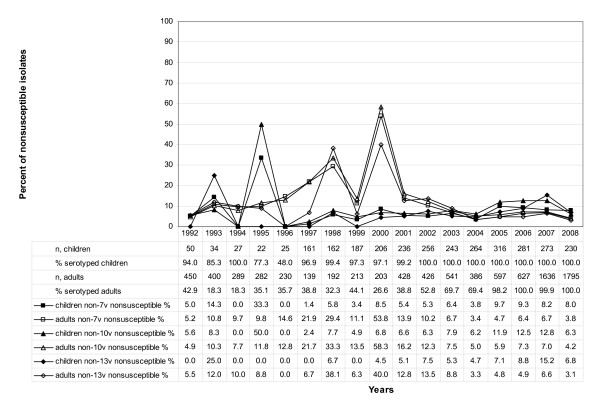
**Macrolide nonsusceptibility among non 7-, non 10- and non 13-valent vaccine serotypes**. Macrolide nonsusceptibility among non 7-, non 10- and non 13-valent vaccine serotypes (IPD isolates in Germany from 1992 to 2008; n, number of cases)

The peak in nonsusceptibility among 7-, 10- and 13-valent serotypes in adults from 1998 to 2002 (Figure 4) correlates to an increased incidence of serotype 14 during that time [10]. Generally, the rate of resistance is higher among the vaccine serotypes (7v, 36.6%; 10v, 28.2%; 13v, 24.3%) (Figure 4) than among the non vaccine serotypes (non 7v, 6.5%; non 10v, 7.4%; non 13v, 6.3%) (Figure 5). The proportion of nonsusceptible 7-valent vaccine serotypes remained largely constant from 2000 to 2007 among children (Figure 4). Among the non PCV7 serotypes the rate of nonsusceptibility is lower (Figure 5). Concerning adults, an increase of isolates sent to the NRCS can be noticed (Figures 4 and 5). The fraction of nonsusceptible isolates has declined during the last years among 7-valent vaccine serotypes after a notable increase from 1992 to 1999 (Figure 4).

## Discussion and conclusions

This paper presents the results of 17 years of surveillance for macrolide susceptibility of invasive pneumococcal disease in Germany. The prevalence of antibiotic-resistant *S. pneumoniae *continues to increase worldwide but varies widely between countries [11-13]. In Europe, high resistance rates for macrolides have been reported from France, Spain, Italy and Belgium [12,13]. Pneumococcal macrolide resistance rates reported from Germany were low [12-17]. Nevertheless, a continuous and statistically significant increase of macrolide nonsusceptibility could be observed after publication of these studies, reaching maximum values in 2005 (children: intermediate, 0.3%; resistant, 32.3%; adults: intermediate, 0.0%; resistant, 18.6%). The relatively high rate of variation in resistance among childhood isolates during the first years of the study is presumably due to the low number of cases, and a suspected bias for resistant isolates among the centers sending the isolates. Since 2005, a considerable and statistically significant decrease especially for childhood nonsusceptibility has been noticed. These data are similar to those recently reported from Spain, where a reduction of erythromycin nonsusceptibility from 42.9% in 2003 to 20.0% in 2007 has been described [18].

An increased awareness of IPD among adults has been observed since 2007. This correlates to the general recommendation of pneumococcal conjugate vaccination for children < 2 years in Germany at the end of July 2006 and an increased interest in serotype information of IPD. Furthermore, in January 2007 an internet based laboratory sentinel system ('PneumoWeb') was established in Germany, which enables participating laboratories to transfer anonymised basic patient information on a voluntary basis. Compared to children, only a minor reduction of nonsusceptibility has been observed among adults from 2005 (18.6%) to 2008 (13.0%), although this reduction was also statistically significant.

Possible reasons for the decrease in macrolide nonsusceptibility include a reduced macrolide consumption due to the rising resistance rates, as well as the general recommendation of pneumococcal conjugate vaccination for children < 2 years in Germany at the end of July 2006. Since the introduction of the vaccine a considerable decrease of serotypes included in the 7-valent pneumococcal conjugate vaccine has been observed among German children, but also (to a lesser extent) among adults [10], which is partly due to the association of serotypes with age [19,20].

The antibiotic prescribing practices, which are thought to be among the most significant drivers for the spread of pneumococcal resistance, differ vastly between European countries [15,21-23]. A decrease in the use of macrolides has been reported for instance in Spain [18], Portugal [24,25], Belgium [26], Slovenia [27] and Taiwan [28,29]. The influence of a decreased macrolide consumption on macrolide susceptibility is discussed controversially. In Spain a relation between the decrease in macrolide consumption and the decrease in erythromycin non-susceptibility among children could be shown, while this effect was absent among the adult population, probably due to the increase in non-vaccine serotypes such as 19A (from 3.6% of all invasive serotypes in 2000 to 10.1% in 2007) [18]. Reports from other countries showed no decrease in macrolide nonsusceptibility following a reduced macrolide consumption [25-29]. Besides the total macrolide consumption, the influence of long lasting macrolides, which may increase even in times of decreasing total macrolide consumption [25], is discussed to be a cause of the macrolide nonsusceptibility [25,30-32]. Besides antibiotics, pneumococcal conjugate vaccination is another important factor associated with changes in macrolide susceptibility [25,26,33-36].

In our study, high rates of serotype specific resistance among the more frequent serotypes were observed among the serotypes 14, 6B, 19F and 23F, in particular. These results are in line with results from Germany published previously by the NRCS for invasive [14] and respiratory tract isolates [37] and comparable to results reported from several European countries [3,13,38,39]. Notably, a statistically significant trend in increasing macrolide resistance was seen for serotypes 14 and 19F. However, since both serotypes are included in the pneumococcal conjugate vaccines, a future reduction of these serotypes can be expected. The low rate of macrolide nonsusceptibility among isolates not serotyped corresponds to the fact, that high resistance levels were a main trigger for initiation of serotyping during the early years of this study, when consistent serotyping of all isolates was not conducted due to excessive costs.

In spite of all these observations, because the impact of preventive and therapeutic strategies on pneumococcal evolution not only depends on, but also influences the serotype distribution, when normal temporal [11,40] and regional [15,41,42] variations of serotype distribution are taken into consideration, future developments remain difficult to predict [32]. Ongoing nationwide surveillance is necessary to observe further developments of pneumococcal macrolide resistance in Germany.

## Authors' contributions

MI performed the analysis and drafted the manuscript. CM performed the statistical analysis. MI, RRR and ML participated in the laboratory analyses. MI, RRR and ML conceived the study. All authors read and approved the final manuscript.
